# The Impact of Peripheral Artery Disease (PAD) on Lower Limb Kinematics in Type 2 Diabetes Mellitus

**DOI:** 10.1900/RDS.2021.17.11

**Published:** 2021-08-01

**Authors:** Claire Saliba Thorne, Erica Bartolo, Alfred Gatt, Cynthia Formosa

**Affiliations:** Faculty of Health Sciences, University of Malta, Msida, Malta.

**Keywords:** kinematics, lower limbs, biomechanics, diabetic foot, ankle joint, knee joint, peripheral artery disease

## Abstract

**BACKGROUND:**

Peripheral artery disease (PAD) and diabetes mellitus are factors known to influence gait characteristics. However, there is a lack of knowledge on the extent to which type 2 diabetes mellitus (T2D) and PAD as comorbidities cause limb and gait complications.

**AIM:**

The purpose of this study was to investigate the impact of PAD as a complication of T2D on ankle joint dorsiflexion and knee joint flexion angles using an optoelectronic motion analysis system and to find out whether these alterations are complications secondary to neuropathy or reduced blood perfusion.

**METHODS:**

Ninety participants were recruited in this quantitative study which applied a prospective, comparative, non-experimental approach. Participants with T2D and PAD (n = 60), categorized according to the severity of PAD (mild and severe group), were compared with a control group consisting of patients with T2D alone. An optoelectronic motion capture system was used to record mean maximum flexion angles of the knee joint and maximum mean dorsiflexion angles of the ankle joint during gait.

**RESULTS:**

180 limbs were analyzed. Both mild and severe PAD participants exhibited a significant increase in mean maximum ankle joint dorsiflexion angles (p = 0.001) and a significant decrease in mean maximum flexion of the knee joint compared with the control subjects (p = 0.001).

**CONCLUSIONS:**

This study shows that T2D and PAD alter ankle joint and knee joint kinematics. This research provides biomechanical understanding of limb and gait alterations in this specific patient population which may contribute to an improved understanding of gait alterations and clinical management. The findings suggest that the reduction in ankle joint dorsiflexion commonly attributed to glycosylation in diabetes may be secondary to neuropathy and not to reduced blood perfusion.

## 1 Introduction

Peripheral artery disease (PAD) is characterized by occlusion of the arteries of the lower extremities [1,2]. Femoral, popliteal, tibial, and peroneal arteries are most commonly affected, leading to a higher risk of lower-extremity amputation [3, 4].

The risk of developing PAD is four times higher in the presence of diabetes mellitus than in its absence. Diabetes is rated as the strongest risk factor for the development of PAD [7-11], with a one percentage rise in HbA1c increasing the risk of developing PAD by 26% [5, 6]. Studies investigating the kinematics of people with PAD and type 2 diabetes mellitus (T2D) as separate morbidities report distinct gait variability, particularly in the ankle joint and knee joint [12].

Patients living with T2D alone tend to exhibit reduced ankle joint dorsiflexion and knee flexion during gait [13-16], particularly in the presence of peripheral neuropathy [17-18]. Apart from T2D, peripheral arterial disease alone was found to affect the ankle joint by increasing dorsiflexion during gait and to reduce knee joint flexion [19-22]. To date, there have been no studies investigating the effect of both PAD and T2D as comorbidities on ankle joint dorsiflexion and knee flexion.

Altered biomechanical conditions brought about by both PAD and T2D may put this patient population at higher risk of developing deformities of the foot structure and muscle weakness, which in turn may result in higher predisposition to tissue stress [23], ulceration [24-26], falls, and even amputations [27]. Studies by Formosa *et al*. highlight the importance of meticulous and in-depth biomechanical examination with particular focus on joint movement and foot deformities [28, 29].

Many studies concentrated on the effect of diabetes on lower limb kinematics, but the impact of PAD is frequently disregarded [13-18]. It is plausible that the previously mentioned alteration in lower limb kinematics is secondary to nerve damage and muscle weakness resulting from long-standing ischemia. Muscle function is greatly dependent on vascular supply and the lack of it may alter the gait.

3D gait analysis may provide new insight into the mechanisms underlying altered biomechanics that may cause an increased risk of foot ulceration in patients with T2D and PAD. Thus, the purpose of this study was to investigate the impact of PAD as a complication of T2D on ankle joint dorsiflexion and knee joint flexion angles utilizing an optoelectronic motion analysis system.

## 2 Methods

Ethical approval was granted by the University Research Ethics Committee and all consenting participants were treated according to the Declaration of Helsinki [30]. Using a purposive sampling technique, 90 participants (180 limbs) aged 50-75 years, presenting with T2D and PAD of varying severity, were recruited from local health centers. Participants with the following conditions were excluded from this study to avoid influences other than PAD on limb complications [12, 20, 22, 31]:

- History of musculoskeletal pathologies such as rheumatoid arthritis (RA), osteoarthritis (OA), and foot deformities
- History of amputation
- Neuromuscular disorders especially Charcot-Marie-Tooth disease
- Peripheral neuropathy

Participants were also excluded if they required walking aids such as crutches, wheelchairs, or prosthesis [12, 20, 22, 31].

In order to ensure the validity, precision, reliability, and integrity of the research study, thorough selection of participants that matched the inclusion criteria was carried out and a representative sample of the general population was recruited. Rigor and prevention of bias were applied throughout the study.

The tools selected to conduct this study, including the neurothesiometer, neuropen, ankle brachial pressure index (ABPI), toe brachial pressure index (TBPI), force plate, and 3D motion capture system, were all certified as reliable and valid and were deemed to be a gold standard tool for the diagnosis of neuropathy, level of PAD, and measurement of the kinetics and kinematics of the human body, respectively.

Prior to recruitment, the participants were screened using a 10g monofilament and a neurothesiometer to exclude the presence of peripheral neuropathy [12, 32, 33]. Presence and severity of PAD were assessed using TBPI, which compares the systolic pressure of the brachial artery with the pressure of the big toe, as per standard protocol [34]. Prior to using neurothesiometer, neuropen, ABPI, and TBPI, the participants were given enough time to acclimatize to the environment and rest for not less than ten minutes.

The participants recruited were categorized into three groups according to their TBPI results:

- Participants with a TBPI greater than 0.7 were classified as T2D without PAD (control group, n = 30).
- Participants with a TBPI between 0.64-0.7 were classified as patients with mild PAD (T2D + mild PAD group, n = 32).
- Participants scoring a TBPI of less than 0.64 were categorized as patients with severe PAD (T2D + severe PAD group, n = 28).

Retroreflective markers were placed on specific anatomical landmarks as instructed by the Plug-In Gait model (Vicon, OMG, Oxford, UK) [35]. Prior to data collection, a static subject calibration was performed to customize a subject skeleton, to define the relationship between each marker, and to permit real-time conversion of images coordinated for each marker versus virtual coordinates captured by each camera [36].

Each participant was asked to walk at a self-selected speed along a 10 meter walkway during which ten infrared cameras captured his motion in the calibrated volume at a rate of 100 Hz. Ten cameras were set up along the laboratory walls to avoid unwanted movement or damage during the procedure, while maintaining enough flexibility to adjust for the position required and avoid ‘dead space’, i.e. any space that does not lie within the field of view of the cameras. The walking trials deemed to be incorrect were discarded, as per standard gait analysis practice. One session (recording) for each participant comprised a total number of 24 trials (walks), which were then all averaged in preparation for statistical analysis and interpretation.

In this study, reliability was ensured by using the same equipment throughout the study to ensure consistency in the results. Thorough attention was also given during data collection to ensure high quality data by paying attention to hardware set-up such as camera settings, capture volume, position and number of cameras, calibration techniques, and marker placement. The system resolution is affected by the capture volume setting, and was thus regulated to compromise between the activity being recorded and the quality resolution of the system.

After the data captured had been processed using Butterworth filtering, maximum angular movement of the ankle joint and that of the knee joint (sagittal plane movement) were recorded, from which mean maximum ankle dorsiflexion angles and knee flexion angles were derived.

The Kolmogorov-Smirnov test was used to verify the distribution of the data. The results were analyzed statistically using one-way ANOVA, while a Tukey’s honest significant difference (HSD) test (as a post-hoc test) was also applied to determine which group exhibited significant differences in the dorsiflexion and flexion angles [37]. All statistical analyses were conducted with the Statistical Package for the Social Services (SPSS, IBM) version [24].

## 3 Results

One-way ANOVA showed a statistically significant difference between the maximum ankle dorsiflexion angle of the control group (T2D only) and participants with both T2D and PAD (p = 0.001, Table 1). A statistically significant difference was also found between the maximum knee flexion angle of the control group and that of the participants with both T2D and PAD (p = 0.001, Table 2).

**Table 1.** One-way ANOVA statistical results for maximum dorsiflexion at the ankle joint

Group	n	Mean angles (degrees)	Std. deviation	p-value
Normal	60	17.22	5.40	0.001*
Mild	64	21.18	4.96	
Severe	56	21.25	5.36	
Total	180	19.46	5.65	<0.001

**Table 2.** One-way ANOVA statistical results for the maximum flexion at the knee joint

Group	n	Mean angles (degrees)	Std. deviation	p-value
Normal	60	45.13	9.91	0.001*
Mild	64	29.67	6.80	
Severe	56	27.38	7.19	
Total	180	34.27	11.34	0.001*

Post-hoc analysis, for both the ankle joint and knee joint, concluded that there were differences in mean scores between both the control versus severe PAD group (difference of 4.04° for the ankle joint dorsiflexion and 17.75° for the knee joint flexion; p = 0.001) and between the control versus mild PAD group (difference of 3.96° for the ankle joint dorsiflexion and 15.46° for the knee joint flexion; p = 0.001, Tables 3 and 4). Both mild and severe PAD groups exhibited an increase in mean angles for both ankle joint and knee during maximum dorsiflexion/flexion.

**Table 3.** Post-hoc statistical results for maximum dorsiflexion at the ankle joint

Groups	Mean difference (degrees)	p-value
Normal vs. severe	4.04	0.001*
Normal vs. mild	3.96	0.007*
Severe vs. mild	0.08	0.998

**Table 4.** Post-hoc statistical results for maximum flexion at the knee joint

Groups	Mean difference (degrees)	p-value
Normal vs. severe	17.75	0.001*
Normal vs. mild	15.46	0.001*
Severe vs. mild	02.29	0.304

## 4 Discussion

This study is the first of the few existing studies [12, 20, 22] to explore the effects of PAD on lower limbs in a population with PAD as a complication of diabetes mellitus. It is also the first to investigate the effects of speed variation on the ankle joint and knee joint of patients with both T2D and PAD.

The results from this study confirm that patients with PAD as a comorbidity of T2D exhibited a greater mean maximum dorsiflexion of the ankle joint and reduced mean maximum flexion angles of the knee joint during the stance phase of gait than those with T2D only.

Previous studies have shown that patients with T2D only exhibiols [19, 22, 37-40]. This study provides novel information relating to the coexistence of these two conditions, which is common in clinical practice. We found that PAD has a greater impact on ankle dorsiflexion than diabetes alone.

Long-standing diabetes mellitus, especially if uncontrolled, is known to be a deteriorative factor on nerves which in turn affect muscle function and gait. Studies investigating kinematics in people with diabetes only observed that these patients exhibited a reduction in ankle joint dorsiflexion and knee flexion during gait [13, 16], particularly in the presence of peripheral neuropathy [17, 18]. Although the control group in the present study achieved low angular measurements of ankle joint dorsiflexion compared to normative expected values, the angular results were slightly higher when compared with those of diabetic participants in other studies. In our study, participants with good glycemic control acted as controls, which emphasizes the importance of well controlled blood glucose levels. It also highlights the influence of glycemic control on the kinetics of the lower limb.

The present study has recruited the largest sample size (n = 90) among those studies investigating the biomechanical alterations in T2D and/or PAD and assessed bilateral limbs (180 limbs in total). The participants in the study group had both T2D and PAD, and were compared with people with controlled T2D alone who acted as controls. To the best of our knowledge, this scenario has never been investigated before.

Even though both groups may have experienced glycosylation of the muscles because of the presence of diabetes, participants with PAD are also likely to exhibit compensation secondary to muscular weakness. As a compensatory mechanism, a reduction in the range of motion of one joint (knee joint) brings about an increase (overcompensation) in range of motion in another joint (ankle joint) with the purpose of maintaining an efficient forward movement [41]. This distinct difference between the effects of the two diseases may be attributed to an adaptation to a biomechanical function, or to a neuromuscular weakness brought about by peripheral arterial disease. Nerve damage and muscle weakness of the anterior, lateral, and posterior compartment of the ankle joint are hypothesized to limit dorsiflexion during gait [42, 43]. Moreover, alterations in lower limb biomechanics may cause an increase in tissue stress, with a consequent increase of tissue breakdown, especially among people with diabetes and PAD [23].

Being the farthest anatomical structure of the body, the calf muscles are more likely to be affected by glycosylation and stiffness caused by prolonged ischemia and diabetes [38, 44]. This may result in an imbalance between the anterior and posterior compartment of the lower limb, causing the foot dorsiflexors to overwork and compensate for the deficient function of the plantar flexors.

Associated deformities of foot structure may cause high risk of foot ulceration in weight-bearing areas [45]. Thus, people with diabetes are at a higher risk of lower-limb amputations than healthy people, especially in the presence of PAD, which accelerates the risk by directly damaging nerves, blood vessels, and collagen synthesis [27].

## 5 Conclusions

People with PAD and T2D were known to have a greater ankle joint dorsiflexion and a reduced knee flexion compared to people living with T2D only. This difference between the two groups may be attributed to a compensatory mechanism during gait. Furthermore, the reduction in ankle joint dorsiflexion presented by the patients with T2D may be primarily the result of early stage neuropathy and not of reduced blood perfusion.

Participants suffering from severe PAD, with a TBPI score of less than 0.64, were found to have a 4.04 degree increase in the ankle joint during maximum dorsiflexion and a 17.75 degree reduced knee flexion compared to people with T2D only. This alteration may be attributed to a mechanism compensating for glycosylation and oxygen deprivation (i.e. ischemia) to the farthest anatomical structure of the body, the calf muscle.

The novel aspect of this study is the fact that the observations made question whether the expected decrease in the ankle joint dorsiflexion, commonly revealed in diabetes, may be secondary to early stage neuropathy and not secondary to reduced blood perfusion alone. Furthermore, the study highlights the importance of having well controlled blood glucose levels which impact the kinematics of the body during gait.

Changes in the biomechanical function of the lower limb, especially in high-risk patients, are known to cause an increase in tissue stress and tissue breakdown, leading to possible amputations and early mortality rates. This study proposes a new perspective for examining the high-risk foot and for better understanding the effects of PAD and diabetes on the lower limb.

## 6 Recommendations

A reduction in knee joint flexion angle was found to increase the risk of falling among participants with PAD [26], which should be investigated in future studies with special focus on patients with PAD as a complication of diabetes mellitus. It is also recommended to explore in more detail the ankle joint regarding neurological and mechanical effects on the angle of this joint in participants with diabetes mellitus and PAD to find out why there may be an increase in joint angle, as shown in this study.

Future studies may reinvestigate in more detail the effect on kinetics and kinematics of a population with combined T2D and PAD at different walking speeds, with the aim of improving the alterations in diabetes-related biomechanics.

Timing of maximum dorsiflexion/flexion and plantarflexion/extension of joints in relation to the phases of the gait cycle would be an interesting and valuable aspect to explore as little research is currently available in relation to this patient population. Such insight would help the clinician to understand better the biomechanical limitations of people with both conditions and to optimize treatments that improve mobility and prognosis in these individuals.

Future studies are also recommended to explore the effect of PAD in the presence of diabetes mellitus on the muscle power and timing of contraction during gait using surface EMG. Lack of vascularization to the muscle is considered to cause a possible reduction in muscle effectiveness which in turn may result in alterations in the range of motion of the joints [20].

## Abbreviations

ABPI ankle brachial pressure index

ANOVA analysis of variance

EMG electromyograph

HSD honest significant difference

OA osteoarthritis

RA rheumatoid arthritis

PAD peripheral artery disease

T2D type 2 diabetes mellitus

TBPI toe brachial pressure index

SPSS Statistical Package of the Social Services
